# Assuming accuracy, pretending influence? Risks of measuring, monitoring and reporting sustainable development goals

**DOI:** 10.1007/s13280-022-01787-z

**Published:** 2022-09-30

**Authors:** Jari Lyytimäki, Nicolas Eckert, Robert Lepenies, Claire Mosoni, Jyri Mustajoki, Anders Branth Pedersen

**Affiliations:** 1 Finnish Environment Institute, Latokartanonkaari 11, 00790 Helsinki, Finland; 2 INRAE, UR ETNA, Université Grenoble Alpes, 2 rue de la papeterie, 38402 St Martin d’Heres, France; 3 Karlshochschule International University, Karlstrasse 26-28, 71633 Karlsruhe, Germany; 4 Finnish Environment Institute (SYKE), Latokartanonkaari 11, 00790 Helsinki, Finland; 5 Department of Environmental Science, Aarhus University, Frederiksborgvej 399, 4000 Roskilde, Denmark

**Keywords:** Agenda 2030, Indicators, Risk management, Risk perceptions, Sustainability

## Abstract

From the local to global level, indicators and reports are produced and published to support the transition towards sustainable development. Building from two European-level science–policy workshops, this perspective essay discusses the types of risks involved with such sustainability reporting. The analysis is rooted in the framework of the UN 2030 Agenda and sustainable development goals (SDG). As a globally adopted framework, it provides an example of how risks are either recognised and framed, or non-recognised. Well recognised risks include data availability for SDGs and siloed preparation of indicators, while risks receiving less attention are ritualistic reporting lacking a critical evaluation of the limitations of the SDG framework itself. These different risks are likely to reinforce each other. A specific risk is a too narrow focus on one-way communication aiming to inform individual policy decisions. Risks related to SDGs are best managed with iterative, integrative and interactive knowledge production fostering holistic understanding.

## Introduction

“I believe that the 2021 monitoring report will inspire European citizens, policy-makers, researchers and businesses to undertake sound sustainable development actions…”.

Mariana Kotzeva, Director-General, Eurostat (Eurostat 2021, p. 5).

Sustainability assessments and reports often express a desire not only to inform but also to inspire wide and effective actions throughout societies (Eurostat 2021; Lafortune et al. 2021). Such a desire is rooted in a technocratic approach to decision-making, assuming that improving the knowledge base, spelling out the evidence and identifying key risks related to environmental degradation will motivate politicians to make necessary decisions (Slovic 2000; Sunstein 2002). However, this approach is challenged by the complexity of sustainability challenges, patchy knowledge and different values and interests by both experts and lay people. Even complete agreement on the characteristics of sustainability issues would not guarantee influence on decision-making. Human behaviour is always influenced by biases, selective use of information and mental shortcuts (e.g. Slovic 2000; Kahneman 2011).

Furthermore, the policy uptake of science advice on sustainability challenges has been challenged by a populist influence (Tumber and Waisbord 2021). Populists tend to distrust experts and argue that in a democracy, government needs to follow the will of the citizens—leading to a risk of non-recognition of potentially flawed perceptions of sustainability issues. The rise of populist movements and government leaders, as well as a broad umbrella of media and social media influencers delivering fabricated news, has highlighted the challenges of distorted and simply wrong data (Tumber and Waisbord 2021).

From the perspective of a sustainability transition, favouring far too simple solutions to complex societal problems is perhaps the greatest risk of populism. Identifying and communicating the best available science-based advice are not simple nor straightforward (Morse 2004; Renn 2008). Various knowledge tools have been developed to summarise and simplify complex issues into messages that can be effectively delivered to and easily digested by various audiences on different levels and sectors of societies. The format of reporting varies from interactive online portals, or concise policy briefs, to thick assessments featuring hundreds of indicators. Reporting processes developed by the International Panel on Climate Change (IPCC) are among the best-known examples of authoritative science-based assessments addressing sustainability issues (Budescu et al. 2012). The IPCC approach is essentially based on the ideal of a technocratic approach of objective calculations for rational policy-making (Tangney 2020). Reporting as part of the Sendai Framework for disaster risk reduction provides another key example of a global initiative.

Despite all efforts, sustainability reporting has so far largely failed in achieving its core mission to inspire and ignite a transformative global change leading to sustainable development. As shown by the inadequate responses to climate threats, societies still have limited capacities to implement science-based recommendations on how to solve comprehensive societal issues (von Stechow et al. 2016). The sustainability indicators and assessments show that the world is not on a path towards sustainable development (OECD 2019; Eurostat 2021; Lafortune et al. 2021). A possible explanation is that the expectations for the capability of sustainability reporting to inspire and encourage action have been too optimistic. From a less ambitious perspective, already reaching a global-level agreement on a holistic list of sustainability goals is a considerable achievement (Messerli et al. 2019).

The risk of overly optimistic expectations has been noted by studies demonstrating that the direct influence of sustainability indicators on policy decisions is typically small (Rinne et al. 2013; Lehtonen et al. 2016). Some studies suggest non-intended effects of reporting and the risk of misuse of indicators (Lyytimäki et al. 2013). However, it should be noted that the long-term and indirect societal influences of sustainability indicators and reporting are notoriously difficult to identify, and it is possible that both positive and negative long-term influences remain hidden.

The general aim of this perspective essay is to illuminate the diversity of risk perceptions related to sustainability reporting. We focus on the framework of the UN Agenda 2030 and the Sustainable Development Goals (SDG) that provides a policy-relevant and widely applicable example. We identify the types of risks perceived as particularly relevant for public and private actors from the SDG perspective and suggest strategies to improve risk management throughout the journey towards sustainability. The essay also aims to complement other studies focusing on assessing and managing risks related to SDGs (Allen et al. 2018; Lyytimäki et al. 2020).

## Background and materials

We combine reflections from academic literature and practitioner insights from two European-level workshops, complemented with national-level experiences from sustainability indicator production and communication processes. Materials were compiled under the project “Research on Sustainable Development Goals: Tackling and managing risks with SDGs (PEER-TRISD)” by the Partnership for European Environmental Research (PEER) combining insights from eight research institutes.

The first workshop, “Risks and sustainable development: Workshop for scanning ways of anticipating, monitoring and governing risks in relation to SDGs”, was organised in Brussels, Belgium, on 22 January 2019. It aimed to connect scholars with leading European representatives of policy, finance, insurance and industry to address the interface of SDGs and risks. Considering intended and unintended impacts of public policies and private activities aimed to achieve SDGs, the 33 participants discussed who are likely to experience risks and who carries the responsibility of managing and governing them.

The second workshop, “Integrating the evaluation and management of risks and sustainable development: a solutions workshop”, was organised online due to the COVID-19 situation. Approximately 60 participants and panellists represented governments, finance, cities, industry, insurance and research. The aim was to discuss evaluation and management of risks and sustainable development. The workshop was arranged on 6 November 2020 in collaboration with European Environmental Evaluators Network Forum 2020. Both workshops acknowledged the polysemous nature of the concept of risk (Kermisch 2012) and aimed to advance a common understanding of the different risk perceptions.

Both workshops followed the Chatham House rules and therefore all insights from the workshop are presented without any personal identifiers. The primary data consist of unpublished workshop reports, personal notes by the organisers and online comments written by the participants of the second workshop. Interpretations presented here are based on the iterative reading of the materials and represent the consensus view of the authors. The key themes emerging from the workshops are discussed in the following sections.

## Risks related to SDG measuring and monitoring

Measuring and monitoring refers here to the processes of data generation and processing supporting the preparation of sustainability indicators and sustainability reporting. Various concerns and risks related to *data quantity* were highlighted in the workshops, as expected based on earlier experiences (Moldan et al. 1997). The complete lack of data, lack of reliable data and unavailability of long-term trend data were emphasised. Quantitatively oriented thinking dominated conceptualisation among the workshop participants. This perception was influenced by the assumption that indicators are essentially based on quantitative time series. This corresponds with the critique of development indicators as a trade obsessed with numbers (Morse 2004). Qualitative indicators were mentioned only occasionally and often indirectly.

National and cross-national *comparability* has been a key concern of SDG indicator development (Allen et al. 2018), but the workshops indicated that a greater challenge may be the incommensurability between public and private sectors. Many industries, banks and insurance companies are among potentially interested users of sustainability information as ESG (Environmental, Social, Governance) reporting becomes mainstream (Jebe 2019). However, even if SDGs are introduced as a common yardstick, it is challenging to find synergies between the data generation streamlined to serve national-level sustainability assessment and data generation serving firm-level needs. There is a risk of using scarce resources for producing non-comparable data. The question is not only how companies can be better equipped to find and use information related to SDGs and translate sustainability risks to investment decisions. It’s also about securing the flow of societally relevant information from the private sector to the public domain, such as data allowing assessment of the attempts of international companies to avoid national taxes (Finér and Ylönen 2017).

Solutions to measurement and monitoring risks are expected from the rapid development and employment of *new data collection and processing tools*. For example, combining information from national registries with satellite data and information from citizen science initiatives may bridge different data needs (Fraisl et al. 2020; Lepenies and Zakari 2021). However, this also creates new questions concerning the ownership of data and privacy, including a growing tension between commercial and privatised data and public open data. Commercial social media applications primarily aimed to collect and monetise user data largely contradict the open data idealism of sustainability reporting and make it harder to maintain easily accessible data repositories.

The problems with data are often about inadequate understanding of what to measure and how to integrate incompatible domains of knowledge in monitoring and reporting (Assmuth and Lyytimäki 2015). SDG monitoring requires integration across multiple policy domains and various temporal, spatial and functional scales. Simply providing more data may make it more difficult to identify reliable and relevant information for data integration. “Scientists can throw a lot [of] evidence [at] people but throwing more may not make a difference”, as noted by one workshop participant. *Maintaining transparency* was highlighted as one key requirement for the successful integration of an increasing amount of different types of data. Transparency is also important for international comparisons as it helps to identify data gaps or the use of unreliable or even false data.

## Risks related to short-termism and siloed reporting

Reporting and communication refers here widely to the knowledge brokerage tools and processes aimed to reach and influence the selected target audience (Godemann and Michelsen 2011; Saarela et al. 2015). These processes are often unpredictable, and dynamics of media, social media and policy debates often cast attention away from early warnings, long-term issues and holistic views of sustainability reporting (EEA 2013). As noted in the workshops, despite the attempts of sustainability reporting to highlight long-term processes, framings of acute crisis and *short-term risks* dominate public debates and construction of risk awareness (Slovic 2000; Kunelius and Roosvall 2021). Dealing with uncertain long-term risks is difficult for democratic governments with short election cycles and for private sector actors concerned with quarterly reporting.

Single risks easily steal attention from holistic considerations focusing on interlinkages across issues, sectors or countries. *Siloed reporting* resulting from a lack of a systemic view was recognised as a key risk. It was stressed that indicators focusing too narrowly on one sector and highlighting relevant but isolated trends can create false positives as development in other sectors remain neglected. Despite the widely accepted goal of policy coherence, “agencies are concerned about one problem and not the big picture”, as noted by a participant in the second workshop. Continuity of integrative mechanisms, such as inter-ministerial committees, was seen as important but often compromised because of changing policy priorities and project-based funding. The workshop participants also acknowledged that because of the risk of losing the appropriate focus of sector-based risk management, it is not possible nor desirable to aim at eradicating all silos.

A cornucopia of *different reporting platforms* was identified in the workshops, but the challenge is to develop tools from different traditions of research and practise speaking the SDG language. Opportunities for learning between different sectors and domains of governance exist and valuable insights can be generated through pilot exercises. The insurance industry was brought up since it deals with risks as its core business. For example, in France a pilot assessment has identified different scenarios on the extent to which French banks and insurance companies are exposed to climate risks in relation to the costs of deviating from the French sustainable development national strategy (ACPR 2020). Other promising approaches include industrial ecology tools, like life cycle analyses, material flow analysis or input–output modelling assessing sustainability of products and systems (Harris et al. 2021). Less attention has been paid to efficient use of the results of these tools. A suggestion from both workshops was the identification and communication of novel or unexpected SDG relations in order to highlight win–win solutions.

Workshop participants presented mixed views on the capability of the *SDG framework to bridge silos*. On the one hand, it was highlighted that SDG indicators on the national levels can make policy-makers accountable. As emphasised based on German experiences in the second workshop, “we finally see that ministries use indicators internally to hold each other accountable”. On the other hand, based on the German experiences it was noted that “there are many promising local initiatives framed in SDG language, but few national policy-makers benefit from using SDG language”. The capability of SDGs to serve as rhetorical tools in communication was considered as poor: “SDGs have a hard time being heard”, as one workshop participant formulated it. Thus, SDGs can be simultaneously considered as concrete and too abstract.

Overall, bridging *different linguistic styles* emerged as a key challenge, since SDGs were perceived as “high-level policy talk” that are hard to translate and apply when, for instance, risks of investments in a funding organisation are appraised. It was noted that due to the lack of monetary indicators, SDGs do not speak “financial language”. Partly because of this, the private sector focusing on firm-level Environmental, Social and Governance (ESG) reporting was seen as distinct from the public sector SDG reporting (see also Jebe 2019). The EU Taxonomy (EU 2020) gradually establishing criteria to determine whether an economic activity is environmentally sustainable was seen as a potential bridge between these approaches. However, on a firm level, the SDGs remain less rigorously defined and more societally oriented than the financially oriented ESG targets. It was suggested that besides common indicators and conceptual development bridging the sustainability and financial risk perspectives, new practice-oriented approaches such as green or social bonds may provide opportunities to connect the two communities better.

## Risk of ritualistic communication

Risk of *ritualistic or symbolic communication* denotes sustainability reporting that aims for a large-scale transformation but makes only nominal societal influence. Such reporting is typically targeted to those already interested in sustainability issues or obliged to follow the reporting and fails to engage with new and wider audiences (Rinne et al. 2013). While such non-use of sustainability indicators and reports was noted in the workshops, also the misuse of sustainability indicators is a growing concern by stakeholders in an age of populism and fabricated news (Tumber and Waisbord 2021). An example of this is the (mis)use of a country's good ranking in international comparisons to justify refraining from more progressive or wide-based actions. In the workshops, a clear demand was to “[m]ake the SDGs a common concern among the bigger audience, instead of only a guide for businesses and policy-makers”. In addition, doubts were presented on the actual impact of SDGs on business and policy decisions, resulting in the risk of so-called rainbow washing (Heras-Saizarbitoria et al. 2022).

The global orientation of the SDG framework increases the risk of non-use or ritualistic use of sustainability indicators on a national level. SDG indicators aimed primarily at facilitating cross-national comparisons are unlikely to optimally meet national-level policy needs. It was suggested in the second workshop that regular parliamentary checks of government action based on locally adapted SDG indicators can help to raise national-level public discussions and improve policy transparency and ambitiousness. Other suggestions included more transparent and participatory evaluations communicated to the UN (Voluntary National and Local Reviews), national budget planning with adapted SDGs and independent evaluations of SDG progress by national audit offices. These could be components of a thorough and action-oriented *risk management* that should be an integral part of implementation of SDGs. However, the SDG framework directs attention more towards ex-post-risk assessment than ex-ante management of risks. In addition, it directs attention to assessing global-level grand challenges rather than management and orchestration of context-specific actions relevant for local actors (Salo et al. 2022).

Ritualistic reporting is partly a result of conceptual frameworks and institutional settings incapable of properly *addressing user needs* in an action-oriented way (Lehtonen et al. 2016; Rinne et al. 2013). As a participant at the second workshop noted: “SDGs are a monumental work, but what is missing is help formulating the actions”. Possible solutions include communication and interaction processes tailored to correspond with specific actors on individual micro level (e.g. the insurer looking at an asset), on the intermediate level (e.g. neighbourhood communities) and on the societal macro level (e.g. public authorities). SDGs have improved possibilities for coherent discussions and policy dialogue by introducing a common terminology. If adequate resources for communication and interaction are available, tailoring the messages, based on an already shared conceptual basis, is relatively easy. The greater challenge lies in reconciling potential discord between different interests and actors. However, this also provides an opportunity for knowledge brokering, patiently bringing together different views under the unifying overall sustainability framework (Saarela et al. 2015).

At the workshops, using national and international *science panels as translators and engagers* was raised as an underutilised opportunity. However, many of the panels have a relatively narrow focus on topics such as climate change. Sustainability-oriented panels with a wide scope often lack resources and a strong institutional position (Kaaronen 2016). It is also questionable whether panels focusing only on science can make a difference. Fact-based reporting is not enough for societal impact, as noted by a workshop participant stressing the “need to touch people's hearts, not only their brains”. Some workshop participants even questioned the value of producing new knowledge, in line with Glavovic et al. (2021), by claiming that the International Panel on Climate Change (IPCC) should have ceased publishing new assessment reports because all relevant evidence justifying immediate climate action already exists. In terms of communication and engagement, narratives are vital as people do not easily connect with data and factual intelligence only (Al-Shaer et al. 2022). This requires the use of engaging storytelling and alternative visions without sliding towards populism and compromising the factual basis of communication.

## SDGs as an operational framework

In sustainability reporting, risks are commonly framed as unwanted future projections of not reaching a specific SDG or target, as exemplified by the OECD assessments measuring distance to the SDG targets (OECD 2019). Various studies address risks related to methodological questions such as the inadequate capabilities of indicators or modelling approaches to describe SDGs (Aly et al. 2022; van Vuuren et al. 2022). Another stream of research shifts the focus from individual goals or targets to the systemic level and addresses risks related to potential trade-offs or missing synergies (Spaiser et al. 2017; Lyytimäki et al. 2021). These studies are motivated by the need to avoid problem-shifting from one area to another by actions taken to attain SDGs. They often point out the in-build tension between environmental goals requiring minimisation of material and energy consumption and socio-economic goals leading to consumption increases. The importance of *identifying interactions between SDGs* and potentials of cross-disciplinary tools to map across the whole SDG matrix was noted also by the workshop participants emphasising the necessity of finding “new avenues to address progress towards SDGs”.

Even cross-disciplinary approaches typically *limit the examination inside the SDG* framework without critically questioning the relevance or ambitiousness of the 2030 targets (Lyytimäki et al. 2021). This can be problematic since SDGs represent a policy compromise resulting from intergovernmental negotiations and they are not likely to fully correspond with key issues and priorities and target-setting by sustainability science (Vinnari and Vinnari 2022). As noted by one workshop participant, declaring SDG winners when all countries are poor performers may create the perception that no action is needed. The comprehensive framework specifically encouraging local-level adaptations lead to risk of losing the comparability and even cherry-picking, highlighting local success stories or hiding difficult topics.

The SDG framework presents a static goal setting for a period of 15 years. This brings with in a risk of *incapability to address emerging issues* (EEA 2013). In a globalised world, an obvious example is the risk of the emergence of a pandemic caused by easily transmissible virus strains such as COVID-19. Such a risk has been well known by health professionals but not fully acknowledged by the SDG framework. Rigid frameworks such as SDGs may forestall actors to “…proactively look at what kinds of risk are we facing”, as noted by a workshop participant.

Scholarly discussions have both welcomed the comprehensiveness of the SDG framework and cautioned that the wide-spanning framework leads to several risks (Spangenberg 2017; Spaiser et al. 2017). A practical risk is that attempts to compile almost 250 official indicators from all nations are likely to fail. More concise indicator sets are needed since collecting comprehensive, timely and reliable indicator data is lacking even in countries with advanced statistical systems (Lyytimäki 2019; van Vuuren et al. 2022). Finding the right *balance between detailed description and a big picture* perspective is essential. Globally, vertical coordination and cooperation, and thereby vertical policy coherence, can sometimes be more challenging than horizontal policy integration. Risk of losing the comparability and, more widely, a common language allowing the diffusion of best practices was highlighted in the workshops because of the multitude of actors involved. The key question is not *who* would be the most suitable actor to coordinate SDGs, but *which* are the best available combinations of actors for collaboration.

## Recommendations for anticipating sustainability surprises

Despite frequent calls for societal change or even a radical sustainability transformation (Lafortune et al. 2021; Messerli et al. 2019), a disagreement among workshop participants existed concerning whether the idea of sustainability equals stability. On the one hand, the SDG framework is perceived to endorse the maintenance of the stability of current socio-ecological systems. On the other hand, SDGs are seen as a programme of change and action towards tangible results that will not be achieved with business-as-usual approaches. Assuming business-as-usual scenario carries a high risk of misguided actions, especially if individual-level motivations for change are considered. Human decisions are influenced by deeply rooted routines, heuristics and socially conditioned earlier experiences assuming continuity (Hukkinen and Huutoniemi 2014). Therefore, it can be difficult to convince people about even apparent and well-known sustainability problems such as increasing losses due to natural hazards associated with global change, as noted by workshop participants.

Risks related to certain SDGs may include indirect and interdependent cascading effects on other SDG targets. These are likely to lead to the most far-reaching risks but also allow for the greatest SDG benefits (Renn et al. 2019). A key task for science, monitoring and reporting is to describe such leverage points or threshold levels leading to radical and potentially permanent changes of system dynamics (Abson et al. 2017). Changes to the physical environment and especially changes of socially perceived priorities pose a challenge to sustainability reporting since data collection and monitoring may not be able to respond to rapidly changing needs. Better capabilities to anticipate and address such sustainability surprises are needed.

**Fig. 1:**
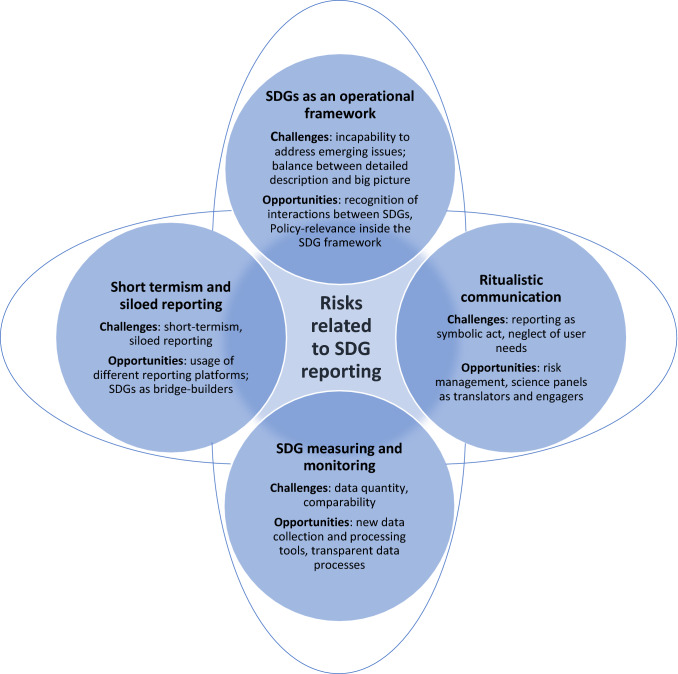
Main themes of SDG risks from the workshops. Risks highlighted by the participants consist of issues related to processes of risk communication (horizontal oval) and issues related to data reliability and framework of knowledge generation and use (vertical oval)

Figure 1 summarises the main challenges and opportunities under four themes identified from the workshops. We conclude with five recommendations for embracing the risks related to SDGs in all their diversity and for anticipating non-linear changes in socio-ecological systems. We would like to stress that while risks generally denote the probability of negative surprises, they also carry information on possibilities for avoiding negative developments or even chances for surprisingly positive changes.

*First,* an obvious lesson is to treat science-based sustainability assessments and indicators in a more realistic way as potentially useful tools with many limitations. Indicators lack capabilities to address many aspects of risks, but they can nevertheless aid decision-making, learning and consensus-building. Utilising the strengths of indicators and minimising their disadvantages requires regular updates, constant development and high transparency.

*Second*, long-term improvement of the already existing monitoring and reporting procedures should be prioritised instead of overly ambitious plans to develop completely new approaches. Making use of the existing knowledge base and complementary knowledge sources, research and practical experience in an inter- and transdisciplinary way is a necessity for sustainable development. As noted by Glavovic et al. (2021), the fundamental problem may not be the lack of knowledge but the brokenness of the science–policy contract, i.e. the expectation that producing more or better knowledge would alone generate action.

*Third,* there is a need to acknowledge the importance of a holistic approach on sustainability both in the private and public sector. This means considering all SDGs: those that are easy to address and excel in, those that are challenging, and those that seem distant or irrelevant. It also means assessing systemic impacts, trade-offs and synergies: the ways in which different SDGs influence each other and can cause cascading risks. Comparisons are needed across multiple domains and temporal and spatial scales. Linking Environmental, Social and Governance (ESG) accounting to official SDG indicators is a promising way for such multi-faceted integration.

*Fourth*, it is important to address the potential built-in flaws or omissions of the SDG framework in forums where policy-makers are held accountable. Specific mechanisms are needed to be able to continuously evaluate SDGs and risks under changing environmental conditions and policy priorities. The line of thinking arguing that SDGs are self-sufficient may lead to a focus on strategies to implement SDGs regardless of the actual changes in socio-ecological systems.

*Fifth,* there is a need to balance knowledge with participation in order to engage a larger audience. This means active engagement of sectors, organisations, targeted sections of organisations, stakeholders, politicians and citizens—especially vulnerable groups and those whose voice is not currently heard. Taking a transdisciplinary approach in research and co-generating knowledge with practitioners may be a fruitful way to produce narratives that can touch peoples' hearts but also be based on robust science.

To summarise, there is a need for an explicit joint agenda for "Risks and SDGs" that helps to bring in risk considerations to the implementation of SDGs and also to the design of the future global sustainability agenda beyond 2030. Strategies embracing rather than avoiding risks should be given primacy. This requires a broad cultural change that goes beyond mere technical improvements of science–policy interactions. As noted by one workshop participant, “managing SDG risks is firstly a systems analysis problem and secondly a human behaviour problem”.
